# Control of Synaptotagmin‐1 Trafficking by SV2A—Mechanism and Consequences for Presynaptic Function and Dysfunction

**DOI:** 10.1111/jnc.16308

**Published:** 2025-01-24

**Authors:** James A. Hogg, Michael A. Cousin

**Affiliations:** ^1^ Centre for Discovery Brain Sciences, Hugh Robson Building, George Square, University of Edinburgh Edinburgh Scotland UK; ^2^ Simons Initiative for the Developing Brain, Hugh Robson Building, George Square University of Edinburgh Edinburgh Scotland UK; ^3^ Muir Maxwell Epilepsy Centre Hugh Robson Building, George Square, University of Edinburgh Edinburgh Scotland UK

**Keywords:** endocytosis, epilepsy, presynapse, SV2A, synaptic vesicle, synaptotagmin‐1

## Abstract

Synaptic vesicle protein 2A (SV2A) is an abundant synaptic vesicle cargo with an as yet unconfirmed role in presynaptic function. It is also heavily implicated in epilepsy, firstly being the target of the leading anti‐seizure medication levetiracetam and secondly with loss of function mutations culminating in human disease. A range of potential presynaptic functions have been proposed for SV2A; however its interaction with the calcium sensor for synchronous neurotransmitter release, synaptotagmin‐1 (Syt1), has received particular attention over the past decade. In this review we will assess the evidence that the primary role of SV2A is to control the expression and localisation of Syt1 at the presynapse. This will integrate biochemical, cell biological and physiological studies where the interaction, trafficking and functional output of Syt1 is altered by SV2A. The potential for SV2A‐dependent epilepsy to be a result of dysfunctional Syt1 expression and localisation is also discussed. Finally, a series of key open questions will be posed that require resolution before a definitive role for SV2A in Syt1 function in health and disease can be confirmed.

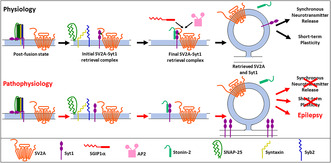

## Graphical abstract

Synaptic vesicle protein 2A (SV2A) is a synaptic vesicle (SV) protein implicated in epilepsy, with a debated physiological role. Emerging evidence suggests that its primary role is the control of presynaptic synaptotagmin‐1 (Syt1) trafficking. Syt1 is a calcium sensor essential for synchronous neurotransmitter release. We propose that SV2A is required for the accurate packaging of Syt1 into SVs during endocytosis, resulting in functional SVs (as depicted in the “Physiology” schematic). SV2A dysfunction results in (1) stranding of Syt1 at the presynaptic plasma membrane and (2) its depletion from SVs (Pathophysiology). This results in disrupted neurotransmission and potentially epilepsy.

**Abbreviations**
AP2: adaptor protein complex 2
GABA: gamma‐aminobutyric acid
GAP‐43: growth associated protein‐43
PI(4,5)P_2_
: phosphatidylinositol (4,5) bisphosphate
PIPKIγ: phosphatidylinositol phosphate kinase 1γ
RRP: readily releasable pool
SGIP1α: SH3‐containing GRB2‐like protein 3‐interacting protein1α
SNARE: soluble N‐ethylmaleimide sensitive factor attachment protein receptor
SV: synaptic vesicle
SV2A: synaptic vesicle glycoprotein 2A
Syt1: synaptotagmin‐1
vGAT: vesicular GABA transporter
vGLUT: vesicular glutamate transporter

## 1 Introduction

Brain function is dependent on communication between neurons that is both rapid and high‐fidelity. Central to this are synapses, which are the fundamental unit of information transfer. The canonical flow of this information is from the presynapse (in the form of activity‐dependent neurotransmitter release) to the postsynapse (which transduces the neurochemical signal into altered excitability). Central to efficient neurotransmission is the coupling of neuronal activity to the fusion of neurotransmitter‐containing synaptic vesicles (SVs) at the presynapse. The protein and lipid composition of a prototypical SV is surprisingly invariant, with multiple studies revealing a tight stoichiometry of cargo proteins (Takamori et al. 2006; Mutch et al. 2011; Wilhelm et al. 2014; Wittig et al. 2021). This stoichiometry appears to be retained between SVs from different neuronal subtypes, with the exception of molecules such as neurotransmitter transporters (Grønborg et al. 2010). This suggests that the specific number of molecules of each independent SV cargo is intrinsic to presynaptic function.

SV cargo must be retrieved from the plasma membrane post‐fusion and formed into SVs by endocytosis. Extensive research over decades has suggested a central role in cargo selection for the adaptor protein 2 (AP2) complex. However, depletion or deletion of any of the genes encoding the subunits of AP2 have relatively minor effects on SV cargo retrieval at the plasma membrane (Kim and Ryan 2009; Willox and Royle 2012; Kononenko et al. 2014; Jung et al. 2015; López‐Hernández et al. 2022). The relatively minor effect indicates that other mechanisms must be present at the presynapse to ensure accurate and efficient SV cargo retrieval. This is the case, with additional cargo adaptor molecules having been identified over the past decade that ensure key players in SV fusion reactions are present on SVs (Jung et al. 2007; Diril et al. 2006; Koo et al. 2011, 2015).

However, the interactions between SV cargo molecules themselves is emerging as potentially the most important determinant of both the inventory and stoichiometry of the SV. A small cohort of SV proteins appear to play essential roles in this regard, which we have previously termed intrinsic trafficking partners (Gordon and Cousin 2016). The abundant SV protein synaptophysin was the founding member. Synaptophysin performs an essential role coordinating the selective retrieval of the soluble N‐ethylmaleimide sensitive factor attachment protein receptor (SNARE) protein synaptobrevin‐2 during SV endocytosis (Gordon, Leube, and Cousin 2011; Gordon and Cousin 2013; Kokotos et al. 2019; Cousin 2021). However, a more enigmatic member is synaptic vesicle glycoprotein protein 2A (SV2A), which shares essential interactions with the calcium sensor synaptotagmin‐1 (Syt‐1) (Yao et al. 2010; Schivell et al. 2005; Schivell, Batchelor, and Bajjalieh 1996; Zhang et al. 2015).

## 2 SV2A‐Dependent Control of Syt1 Function

**FIGURE 1:**
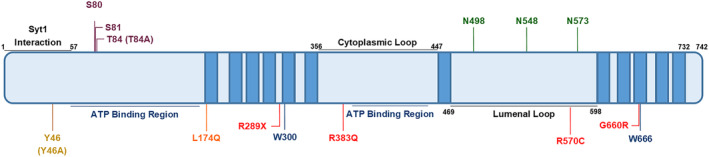
Key amino acid residues of physiological relevance within synaptic vesicle glycoprotein protein 2A (SV2A). Highlighted sites involved in the phosphorylation‐dependent binding of SV2A to Syt1 are displayed, accompanied with mutation (T84A) that perturbs the interaction (purple). Y46 is a residue critical for SV2A retrieval during SV endocytosis, with a mutant version (Y46A) incompetent for retrieval (yellow). Important glycosylated residues of SV2A are also highlighted (N498, N548 and N573: green) thought to be required for botulinum neurotoxin binding. Tryptophan residues proposed to be important for maintenance of neurotransmission (W300 and W666) are indicated in blue. Pathogenic mutations observed in humans with intractable epilepsy (R289X, R383Q, R570C and G660R) are highlighted in red. Finally, a spontaneous mutation that results in epilepsy in a rodent model is indicated (L174Q, orange).

SV2A is a multi‐pass transmembrane SV protein which is expressed ubiquitously in the central nervous system. It is part of a larger gene family, which encompasses brain‐specific (SV2B) and minor isoforms (SV2C) (Buckley and Kelly 1985; Bajjalieh et al. 1994). SV2A has an obligatory role in brain function in rodents, since deletion of the *Sv2ar* gene results in death within 3 weeks following severe seizures (Crowder et al. 1999; Janz et al. 1999). It has a large cytoplasmic N‐terminus which shares interactions with a number of proteins and is also the major site for its phosphorylation (Zhang et al. 2015; Pyle et al. 2000). It also has a large cytoplasmic loop and short cytoplasmic C‐terminus of unknown function (Figure 1). Finally, it has a large lumenal loop that is heavily glycosylated (Kwon and Chapman 2012) (Figure 1). While it is stated above that the number of SV cargo molecules is surprisingly invariant, SV2A is the only protein where there is discussion surrounding its copy number on SVs. The original inventory of SV cargo proteins performed two decades ago suggested that there were only 1–2 copies present per SV (Takamori et al. 2006), whereas subsequent studies have suggested a copy number of between 14 and 17 (Mutch et al. 2011; Wilhelm et al. 2014; Wittig et al. 2021).

The revelation that SV2A is one of the more abundant SV molecules suggests that it performs a key role in presynaptic function, however there is still considerable debate regarding its precise role(s). SV2A has a series of historically proposed functions at the presynapse, ranging from a transporter of ions, neurotransmitters or glucose, to contributing toward the gel matrix that exists inside SVs. These functions are comprehensively summarised in a number of other review articles and will not be covered here (Mendoza‐Torreblanca et al. 2013; Gordon and Cousin 2016; Bartholome et al. 2017; Stout et al. 2019; Rossi et al. 2022). In summary however, there is little evidence that SV2A drives the transport of molecules across membranes. Instead, it appears to perform a key role in the trafficking and expression of the calcium sensor for synchronous neurotransmitter release—Syt1.

### 2.1 The SV2A—Syt1 Interaction

The major interaction partner of SV2A is Syt1 (Schivell, Batchelor, and Bajjalieh 1996; Schivell et al. 2005; Zhang et al. 2015). Syt1 synchronises SV fusion to activity‐dependent calcium influx at the presynapse by binding to calcium via two low affinity C2 domains (Brose et al. 1992; Geppert et al. 1994). The binding of calcium to localised negative charges on the Syt1 C2 domain facilitates binding to acidic phospholipids on the plasma membrane, such as phosphatidylinositol (4,5) bisphosphate (PI(4,5)P_2_) (Brose et al. 1992). This localisation permits the penetration of hydrophobic residues within the C2 domains into the plasma membrane to drive SV fusion (Bai, Wang, and Chapman 2002; Martens, Kozlov, and Mcmahon 2007; Hui et al. 2009). More recent studies suggest that Syt‐1 integrates this function with a series of calcium‐regulated interactions via a specific interface with the SNARE complex, which optimises its orientation to synchronise SV fusion (Toulmé et al. 2024). Furthermore, Syt1 also clamps spontaneous neurotransmitter release and has roles in SV priming (Geppert et al. 1994; Toulmé et al. 2024). Due to these key roles performed by Syt1 in neurotransmitter release, the control/disruption of its expression and trafficking has the potential to have profound physiological and pathophysiological consequences.

SV2A interacts with Syt1 via two independent interaction sites. The best characterised is the interaction of the calcium‐binding C2B domain of Syt1 with the cytoplasmic N‐terminus of SV2A (Schivell, Batchelor, and Bajjalieh 1996; Schivell et al. 2005; Zhang et al. 2015). However, an additional undefined interaction site is present within SV2A, since removal of the N‐terminus still permits an interaction with full‐length Syt1 (Schivell et al. 2005). The interaction with Syt1 is regulated by both calcium and phosphorylation of SV2A. For the former, low micromolar amounts of calcium are proposed to negatively regulate the SV2A‐Syt1 interaction, however the exact mechanism for this control has still to be determined. The first 57 amino acids of the SV2A N‐terminus appear to be important however, since their removal greatly reduces the ability of calcium to inhibit this interaction (Schivell et al. 2005). In addition to calcium, SV2A binding to Syt1 can be regulated via phosphorylation of its cytoplasmic N‐terminus. Initial studies revealed that endogenous phosphorylation of SV2A could be inhibited by antagonists of casein kinase 1 (Pyle et al. 2000). Subsequent work identified the *in vivo* sites on the SV2A N‐terminus that were phosphorylated by casein kinase family members. Two clusters of phosphorylation were identified (Ser42, Ser45, Ser‐47 and Ser‐80, Ser‐81, Thr‐84), however Thr‐84 proved to be essential in facilitating binding of SV2A to a basic patch of amino acids on the C2B domain of Syt1 (Zhang et al. 2015) (Figure 1). Therefore, there is scope for bidirectional modulation of the SV2A—Syt1 interaction by either calcium or SV2A phosphorylation, suggesting this binding is transient and required at specific stages of the SV life cycle.

### 2.2 Physiological Role of the SV2A—Syt1 Interaction

Numerous studies have identified SV2A as a key molecule in the trafficking and expression of Syt1. For example, SV2A knockout brain lysates, neurons and SVs all display a selective reduction in Syt1 expression (Yao et al. 2010; Kaempf et al. 2015). Furthermore, subsequent studies revealed that Syt1 levels were selectively reduced at nerve terminals where SV2A was depleted using shRNA (Harper et al. 2020). However, the major role for SV2A appears to be the retrieval and targeting of Syt1 to SVs during endocytosis. For example, Syt1 accumulates at the presynaptic plasma membrane in either SV2A knockout neurons (Yao et al. 2010) or neurons depleted of SV2A (Zhang et al. 2015; Kaempf et al. 2015). The stranding of Syt1 at the plasma membrane was retained when SV2A mutants deficient for either SV2A internalisation (Yao et al. 2010) or Syt1 binding (Zhang et al. 2015) were expressed in SV2A knockout/knockdown neurons. This indicates that an interaction with SV2A is required for the efficient sorting of Syt1 to SVs from the presynaptic plasma membrane.

Increased plasma membrane stranding of SV cargo is typically used as an indication of dysfunctional retrieval during SV endocytosis (Kim and Ryan 2009; Willox and Royle 2012; Kononenko et al. 2014; Voglmaier et al. 2006; Koo et al. 2011, 2015; Foss et al. 2013; Yao et al. 2010; Gordon, Leube, and Cousin 2011). Paradoxically, the activity‐dependent retrieval of Syt1 is accelerated in the absence of SV2A (Kaempf et al. 2015; Zhang et al. 2015; Harper et al. 2020; Small et al. 2024). This acceleration of Syt1 retrieval does not appear to be due to a global effect on SV endocytosis, since other SV cargoes were internalised efficiently (Yao et al. 2010; Zhang et al. 2015). However, a different study suggested a more global impact on SV endocytosis (Kaempf et al. 2015). Furthermore, overexpression of SV2A appears to both decrease the surface expression of Syt1 (Zhang et al. 2015) and retard Syt1 retrieval, suggesting a reciprocal relationship (Bae et al. 2020). Therefore, loss of SV2A function results in Syt1 accumulation at the plasma membrane, but also an acceleration of its retrieval—a paradox which is discussed further below.

An interesting and related question is, if SV2A controls Syt1 retrieval, what controls the retrieval of SV2A? SV2A has a series of tyrosine‐based internalisation motifs that are recognised by μ‐homology domains, such as that found in AP2 (Figure 1). When one of these key internalisation motifs is mutated (Y46A), SV2A displays considerable surface accumulation (Yao et al. 2010). Furthermore, this Y46A mutant demonstrates greatly retarded activity‐dependent retrieval from the plasma membrane (Small et al. 2024). Therefore, an interaction with a μ‐homology domain‐containing adaptor protein is required for its internalisation. Importantly in the context of Syt1 trafficking, expression of the Y46A mutant in SV2A knockdown neurons retarded the retrieval of Syt1, an effect that is lost when its phosphorylation site is mutated to ablate Syt1 binding (Y46A/T84A mutant) (Small et al. 2024). This is the first direct evidence that SV2A is required for the retrieval of Syt1.

### 2.3 Other Syt1 Trafficking Partners

How could this paradox of accelerated Syt1 retrieval coupled to increased surface stranding occur? It is important to consider that SV2A is not the only molecule that controls Syt1 retrieval during SV endocytosis. Historically, AP2 was considered to be the master coordinator of Syt1 retrieval. However, this is looking increasingly unlikely, even though a series of early biochemical studies suggested that this interaction would be essential. For example, Syt1 has a well characterised interaction with the cargo‐binding μ2 subunit of AP2 within the same patch of basic residues required for SV2A binding (Zhang et al. 1994; Chapman et al. 1998; Haucke et al. 2000). Furthermore, SV2A, AP2 and Syt1 were found to be co‐immunoprecipitated from mammalian brain (Haucke and De Camilli 1999), with multimerization of Syt1 C2B domains increasing AP2 affinity (Grass et al. 2004). Finally, tyrosine‐based endocytosis motifs, including those from SV2A, increased AP2 extraction from brain cytosol by the Syt1 C2B domain (Haucke and De Camilli 1999; Haucke et al. 2000; Grass et al. 2004). All of these studies suggested an essential role for AP2 in Syt1 retrieval. However, a recent study that examined the impact of genomic knockout of the μ2 subunit of AP2 on six independent SV cargoes appears to disprove this (López‐Hernández et al. 2022). In this study, knockout of AP2 retarded the retrieval of synaptophysin, synaptobrevin‐2, the vesicular glutamate transporter (vGLUT) and the vesicular gamma‐aminobutyric acid (GABA) transporter (vGAT), but intriguingly not Syt1 or SV2A. Therefore, it appears the activity‐dependent retrieval of these interaction partners is facilitated by different adaptor molecules.

Other Syt1 adaptors have now been identified, with efficient retrieval of Syt1 dependent on both the monomeric adaptor protein stonin‐2 and SH3‐containing GRB2‐like protein 3‐interacting protein1α (SGIP1α). Intriguingly, both stonin‐2 and SGIP1α have μ‐homology domains very similar to that found in AP2 (Diril et al. 2006; Jung et al. 2007; Kononenko et al. 2013; Lee et al. 2019). However, in contrast to AP2, stonin‐2 interacts primarily with the Syt1 C2A domain, although both C2 domains are required for optimal binding (Jung et al. 2007). Interestingly, the impact of deleting the *Stn* *2* gene results in a very similar phenotype to that observed on SV2A removal, with plasma membrane accumulation of Syt1 and a concomitant acceleration of Syt1 retrieval (Kononenko et al. 2013). Furthermore, overexpression of stonin‐2 reduced plasma membrane levels of Syt1 two‐fold (Diril et al. 2006; Jung et al. 2007)—mimicking the bidirectional control of Syt1 expression observed on manipulation of SV2A. Importantly, this impact on Syt1 retrieval is additive to that observed in the absence of SV2A, since SV2A knockdown in stonin‐2 knockout neurons further accelerated Syt1 retrieval, while exacerbating Syt1 surface accumulation (Kaempf et al. 2015). Therefore, it appears that SV2A and stonin‐2 work via mutually exclusive pathways to sort Syt1 at the presynapse, but with the same functional endpoint.

SGIP1α is the latest molecule to be identified as a regulator of Syt1 trafficking (Lee et al. 2019). As outlined above, it has a μ‐homology domain which interacts with the C2 domains of Syt1; however the exact location of the binding site has yet to be determined. Depletion of SGIP1α in cultured neurons selectively disrupted the activity‐dependent retrieval of Syt1, but not the SV cargoes synaptophysin or vGLUT, with a non‐Syt1 binding mutant of SGIP1α unable to restore Syt1 retrieval (Lee et al. 2019). Interestingly, the surface fraction of Syt1 was unaltered in SGIP1α knockdown neurons (Lee et al. 2019), in contrast to the increased plasma membrane stranding on loss of function of either SV2A or stonin‐2. Therefore, while the Syt1 C2 domains share interactions with SV2A, stonin‐2 and SGIP1α, there is divergence in their functional impact on Syt1 trafficking.

**FIGURE 2:**
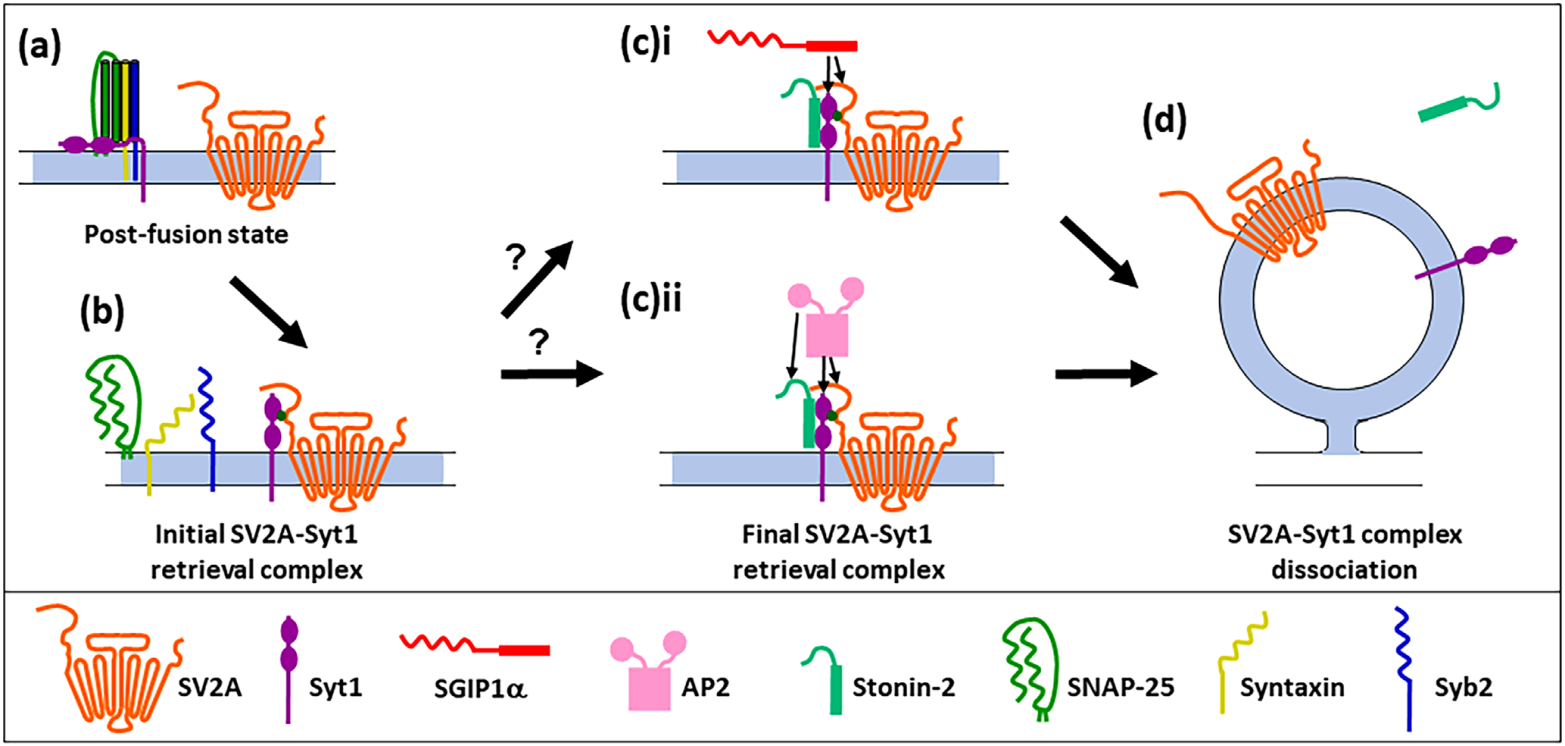
Proposed mechanism of synaptic vesicle glycoprotein protein 2A (SV2A)‐dependent Syt1 retrieval during synaptic vesicle (SV) endocytosis. (a) After SV fusion and neurotransmitter release, all SV cargoes are present on the presynaptic plasma membrane, including the cis‐SNARE complex with Syt1 attached. (b) The cis‐SNARE complex is then broken apart freeing Syt1. Simultaneous to this, the N‐terminus of SV2A is phosphorylated by casein kinase I family members, facilitating an interaction with the C2B domain of Syt1 (Initial SV2A‐Syt1 retrieval complex). (c) Stonin‐2 interacts with the C2 domains of Syt1 to create the final SV2A‐Syt1 retrieval complex. The complex is then clustered for retrieval via the μ‐homology domain of either SGIP1α (c(i)) which shares interactions with Syt1 and possibly SV2A, or AP2 (c(ii)), which has interactions with all three molecules within the final retrieval complex. (d) On formation of the nascent SV, SV2A is dephosphorylated, freeing Syt1 to perform its essential role in triggering synchronous neurotransmitter release. Disruption of this retrieval process may result in disrupted neurotransmitter release, short‐term plasticity and potentially culminates in epilepsy.

One interpretation of the results outlined above is that both SV2A and stonin‐2 act to anchor Syt1 at the plasma membrane, whereas SGIP1α is required for its efficient retrieval (Figure 2). Is there evidence to support this hypothesis? Recent single particle tracking studies suggest that SV2A restricts the movement of Syt1 at the presynaptic plasma membrane, since either Syt1 mutants that cannot bind SV2A or depletion of SV2A with shRNA, both increased Syt1 mobility (Small et al. 2024). Reciprocally, Syt1 mobility at the plasma membrane also increased in SV2A‐depleted neurons in which the non‐Syt1 binding mutant T84A was expressed, but not an internalisation‐deficient form of SV2A (Small et al. 2024). Therefore, SV2A may restrict Syt1 movement to specific regions of the presynaptic plasma membrane potentially to facilitate their co‐retrieval.

### 2.4 Integration of Syt1 Trafficking Partners

The discovery of additional Syt1 trafficking partners has not resolved the paradox that in the absence of SV2A (and stonin‐2), Syt1 retrieval from the plasma membrane is accelerated in spite of increased surface stranding. One suggestion is that increased surface Syt1 acts to accelerate SV endocytosis. Evidence that supports this hypothesis is emerging. For example, it has been acknowledged for a number of years that removal of Syt1 retards SV endocytosis (Littleton et al. 2001; Nicholson‐Tomishima and Ryan 2004; Yao et al. 2011; Bolz et al. 2023). Furthermore, fusion of the cytoplasmic region of Syt1 to the plasma membrane protein growth associated protein‐43 (GAP‐43), results in SV endocytosis acceleration (Bae et al. 2020). However, it should be noted that similar experiments in wild‐type neurons in which expression of GAP‐43 fused to the C2A/B domains of Syt1, had no impact on SV endocytosis kinetics (Yao et al. 2011).

Regardless, a recent study has provided a mechanism for the potential acceleration of SV endocytosis by surface Syt1. This work demonstrated that increased surface expression of Syt1 accelerated SV retrieval via the recruitment of the PI(4,5)P_2_‐generating enzyme phosphatidylinositol phosphate kinase Iγ (PIPKIγ). This localised production of plasma membrane PI(4,5)P_2_ was proposed to facilitate SV endocytosis via the recruitment of the essential fission molecule dynamin‐1 (Bolz et al. 2023). In agreement, knockdown of SV2A results in increased recruitment of dynamin‐1 to the plasma membrane during endocytosis (Small et al. 2024). Therefore, the surface stranding of Syt1 observed in the absence of either SV2A or stonin‐2 may result in a general acceleration of SV endocytosis. This hypothesis also fits with the lack of acceleration in SGIP1α knockout neurons, since SGIP1α knockdown had no discernible impact on Syt1 surface levels (Lee et al. 2019).

## 3 Functional Consequences of SV2A‐Dependent Syt1 Trafficking

Multiple studies have corroborated that SV2A and Syt1 interact, and that SV2A controls Syt1 trafficking. However, what is the physiological consequence of this interaction? Initial studies in SV2A knockout mice hinted at a role in neurotransmission. In addition to defects in evoked neurotransmitter release, SV2A knockout synapses displayed altered short‐term synaptic plasticity, specifically synaptic depression coupled with increased facilitation during action potentials trains (Janz et al. 1999; Crowder et al. 1999; Chang and Sudhof 2009; Nowack et al. 2010; Kaempf et al. 2015). The mechanism of action of this depression is still debated, with some groups proposing a direct control of short‐term plasticity by SV2A via the regulation of residual intracellular calcium (Janz et al. 1999; Chang and Sudhof 2009; Wan et al. 2010), with others postulating a role for SV2A in the size of the readily releasable pool (RRP) (Custer et al. 2006; Xu and Bajjalieh 2001). More recent studies that simultaneously monitored activity‐dependent changes in presynaptic calcium and neurotransmitter release suggest SV2A performs a calcium‐independent role after SV docking (Bradberry and Chapman 2022).

Could SV2A‐dependent Syt1 trafficking potentially underlie any these phenotypes? In support, overexpression of SV2A in wild‐type autaptic hippocampal neurons reproduced the short‐term plasticity defect in neurotransmission observed in SV2A knockout mice (Nowack et al. 2011). This is consistent with a defect in Syt1 trafficking, since overexpression of SV2A limits Syt1 retrieval during endocytosis (Bae et al. 2020). Furthermore, impairments in synaptic strength and short‐term plasticity in SV2A/SV2B double knockout neurons are exacerbated by deletion of the *Stn* *2* gene (Kaempf et al. 2015). Therefore, there is circumstantial evidence that decreased levels of Syt1 on SVs via disruption of SV2A function may result in neurotransmission defects.

However, a limitation of the studies described above is that there has been surprisingly little information relating to the impact of reduced Syt1 levels on its presynaptic role. One study did address this question in autaptic cultures from either Syt1 knockout, heterozygous or wild‐type neurons with Syt1 levels additionally titrated using siRNA (Bouazza‐Arostegui et al. 2022). The results were revealing in that, SV priming and the size of the RRP were exceptionally resilient to loss of Syt1. However, in contrast, clamping of spontaneous neurotransmitter release was perturbed in Syt1 heterozygous neurons. Intriguingly, the most sensitive function to Syt1 expression levels was evoked neurotransmission and release probability, with considerable effects observed even in heterozygous Syt1 knockout neurons (Bouazza‐Arostegui et al. 2022). Finally, short‐term plasticity was also affected in heterozygous Syt1 knockout neurons, in a similar manner to that observed in SV2A knockout circuits. Therefore, the concomitant reduction in Syt1 expression and/or its mistargeting to SVs, that occurs on loss of SV2A function may result in altered neurotransmitter release and short‐term plasticity.

Does evidence exist that the reduced expression and altered trafficking of Syt1 in the absence of SV2A results in the phenotypes outlined above? This is indeed the case, with a decrease in evoked EPSCs and release probability reported in SV2A knockout neurons (Custer et al. 2006), which have a very similar short‐term plasticity phenotype to heterozygous Syt1 neurons (Custer et al. 2006; Janz et al. 1999; Chang and Sudhof 2009). However, it should be noted that dysfunctional synaptic facilitation observed in SV2A knockout neurons could be fully corrected by an SV2A mutant lacking the Syt1‐binding N‐terminus (Chang and Sudhof 2009), a result that may be explained by the additional Syt1 interaction site on SV2A (Schivell et al. 2005). Therefore, it is still unclear whether the sorting of Syt1 to SVs via SV2A is essential for normal presynaptic function and if not for which aspects of neurotransmitter release it is required.

## 4 Consequences of Dysfunctional SV2A‐Dependent Syt1 Trafficking

### 4.1 SV2A Loss of Function and Epilepsy

A key downstream consequence of SV2A dysfunction in rodents is epilepsy. As discussed, SV2A knockout mice experience severe and fatal seizures from 2 to 3 weeks of age (Janz et al. 1999; Crowder et al. 1999). Furthermore, a spontaneous missense mutation in the *Sv2al* gene (L174Q) results in increased seizure susceptibility in rats (Tokudome et al. 2016) (Figure 1). A link to human epilepsy as a result of SV2A dysfunction has been assumed for at least two decades, since the leading anti‐seizure medication, levetiracetam, was found to bind SV2A (Lynch et al. 2004). However, it was not until the identification of mutations in the human *SV2A* gene that a direct link to its loss of function was realised. The mechanism regarding how these mutations culminate in epilepsy is currently unknown however, as is the mechanism of action of levetiracetam, an open question which will be discussed in the Perspectives section.

The first human *SV2A* mutation linked to epilepsy was reported in 2015, in an individual with intractable epilepsy. The individual was homozygous for a missense mutation in the large cytoplasmic loop of SV2A (R383Q) (Serajee and Huq 2015) (Figure 1). After discovery of this initial mutation, two other heterozygous missense mutations (R570C and G660R) were identified (Wang et al. 2019; Calame, Herman, and Riviello 2021; Badura‐Stronka et al. 2023). R570C was located within the large lumenal loop of SV2A, whereas G660R was situated in the 10th transmembrane domain of SV2A (Figure 1). Finally, a homozygous truncating mutation (R289X) was identified in an individual with intractable epilepsy (Al‐Maawali et al. 2024) (Figure 1). Importantly, the discovery of pathogenic mutations in the human *SV2A* gene provides the opportunity to determine disease mechanism. The homozygous R289X mutation may not provide significant information, since it is likely the transcripts will be subject to nonsense mediated decay with the individual effectively knockout for the *SV2A* gene. However, the fact that this homozygous nonsense mutation is not lethal, suggests a form of functional redundancy in humans for SV2A that is not present in rodents.

### 4.2 SV2A Dysfunction and Relationship to Syt1

Intriguingly, there are potential links between SV2A dysfunction, epilepsy and Syt1. For example, SV2A levels are significantly lower in humans with multiple forms of epilepsy and in a series of preclinical epilepsy models. This loss of SV2A is usually accompanied by a concomitant lowering of Syt1 expression (reviewed in (Bartholome et al. 2017)). Further evidence of a potential link between SV2A loss of function, dysregulated Syt1 trafficking and epilepsy comes from the only human SV2A missense mutation to be investigated in detail (R383Q). This mutant displayed increased plasma membrane accumulation, but no overt activity‐dependent trafficking defect (Harper et al. 2020). It also exhibited reduced binding to Syt1, even though the mutation was located in a different region of the protein. This loss of function was reflected in the inability of the R383Q mutant to correct both Syt1 expression and trafficking in SV2A knockdown neurons (Harper et al. 2020). Interestingly, reduced Syt1 expression was also observed in homozygous L174Q rats, suggesting dysregulation of Syt1 function may contribute to observed phenotypes (Tokudome et al. 2016). The remaining heterozygous mutations remain to be investigated. These mutations reside close to residues required for glycosylation and optimal neurotransmission (Dong et al. 2008; Nowack et al. 2010; Chang and Sudhof 2009; Kwon and Chapman 2012), suggesting this may be responsible for the observed dysfunction.

How could altered Syt1 expression/trafficking potentially result in epilepsy? One potential explanation is an imbalance in SV2A expression between excitatory and inhibitory neurons. While SV2A is expressed ubiquitously, it is expressed at higher levels (approximately 20%) in inhibitory terminals (Bae et al. 2020). Importantly, SV2A is the only SV2 isoform expressed in inhibitory neurons, in contrast to excitatory neurons which express both SV2B and SV2C (Bajjalieh et al. 1994). Interestingly, cell‐specific expression of endocytosis reporters revealed that SV endocytosis is slower in GABAergic neurons compared to excitatory neurons across a range of stimulus intensities (Bae et al. 2020). SV2A was implicated in this slowing, since its depletion in inhibitory neurons resulted in a larger acceleration of SV endocytosis than an identical manoeuvre in excitatory neurons. Furthermore, less Syt1 was present at the plasma membrane in inhibitory neurons compared to excitatory counterparts (Bae et al. 2020). Ablation of the SV2A interaction site on Syt1 normalised the difference in its retrieval kinetics between inhibitory and excitatory neurons, confirming the regulation of its trafficking was via SV2A (Bae et al. 2020). However, the notion that SV2A is always expressed at higher levels in inhibitory interneurons may be an oversimplification. This is because SV2A displays distinct expression profiles between excitatory and inhibitory neurons across different hippocampal layers in rodent early development (Vanoye‐Carlo and Gómez‐Lira 2019). Furthermore, this is dynamic, with changes in both SV2A expression and localisation across hippocampal regions in the first month of life (Vanoye‐Carlo and Gómez‐Lira 2019). Therefore, any interplay between SV2A levels at both the cell autonomous and circuit level is likely to be highly complex.

### 4.3 SV2A Dysfunction, Syt1 and Levetiracetam

Levetiracetam is the most widely used anti‐seizure medication in the treatment of epilepsy; however its mechanism of action is still unknown. Therefore, could levetiracetam operate via modulation of the SV2A interaction with Syt1? Early studies confirmed that the presence of SV2A was essential for its therapeutic action, since lowering the *Sv2a* gene dosage in mice reduced the efficacy of the drug (Kaminski et al. 2009). Furthermore, an acceleration of short‐term depression at hippocampal excitatory synapses in the presence of levetiracetam is absent in SV2A knockout mice (García‐Pérez et al. 2015). The acceleration of synaptic depression is consistent with a slowing in SV endocytosis (Ivanova et al. 2021; Chen et al. 2003; Koh, Verstreken, and Bellen 2004; Shupliakov et al. 1997; Koo et al. 2015), however this would not be predicted to result from inhibition of the SV2A‐Syt1 interaction, since increased surface stranding of Syt1 is proposed to accelerate SV endocytosis (Bolz et al. 2023).

**FIGURE 3:**
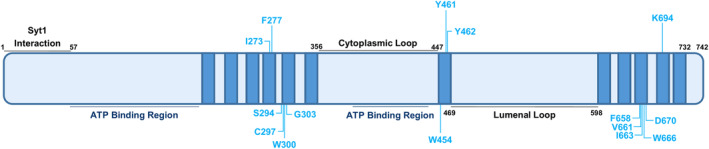
Key amino acid residues implicated in racetam binding to synaptic vesicle glycoprotein protein 2A (SV2A). Residues demonstrated to be involved in racetam binding via either *in silico* modelling, binding assays or a combination of both that are conserved between rat and human models are highlighted. Contributing studies for this figure were: (Shi et al. 2011; Correa‐Basurto et al. 2015; Lee et al. 2015; Mittal et al. 2024; Yamagata et al. 2024; Wood et al. 2018).

A number of binding studies have been performed to determine the interaction site for levetiracetam on SV2A, with a diffuse interaction pocket distributed on the lumenal surface of the SV2A transmembrane domains (Shi et al. 2011; Correa‐Basurto et al. 2015; Lee et al. 2015) (Figure 3). The recent reporting of the crystal structure of SV2A and SV2B with levetiracetam corroborates previous biochemical studies, suggesting that it should not modulate Syt1 binding to SV2A (Mittal et al. 2024). This was confirmed in the same study, since the association of fluorescent‐tagged full‐length versions of SV2A and Syt1 was not altered in the presence of the drug, but was negatively regulated by calcium (Mittal et al. 2024). Therefore, it is unlikely that the therapeutic action of levetiracetam originates from control of SV2A‐Syt1 interaction. A more likely mechanism is that the drug exploits the activity‐dependent sampling of the cell surface by SV2A as a mechanism of uptake, since its presynaptic action appears to be use‐dependent (Yang, Weisenfeld, and Rothman 2007; Yang and Rothman 2009; Meehan et al. 2011, 2012).

## 5 Perspectives

The discovery that Syt1 trafficking is coordinated by SV2A, and that the process is regulated by physiological modulators such as calcium and protein phosphorylation, suggested that it may perform a key role in brain function and potentially dysfunction. Strands of tantalising evidence suggest potential roles and a series of open questions relating to function which are outlined below.

### 5.1 What Controls SV2A Retrieval?

SV2A controls the activity‐dependent trafficking of Syt1 in a bidirectional manner. However surprisingly, it is still unclear whether this trafficking relationship is reciprocal. Furthermore, the identity of the adaptor molecule(s) that mediates SV2A retrieval remains to be determined, since it appears that AP2 performs no role in this regard (López‐Hernández et al. 2022). A key candidate is SGIP1α, since the N‐terminal tyrosine interaction motif on SV2A should interact with its μ‐homology domain. In support, mutation of this motif retards Syt1 retrieval (Yao et al. 2010; Small et al. 2024) in a similar manner to knockout of SGIP1α (Lee et al. 2019). It will therefore be essential to determine whether SGIP1α also controls the retrieval of SV2A.

### 5.2 SV2A in Inhibitory Neurons

Recent studies have revealed that inhibitory neurons contain a disproportionate amount of SV2A compared to excitatory neurons (Bae et al. 2020). This is in addition to SV2A being the only SV2 isoform being expressed (Bajjalieh et al. 1994). The increased levels are proposed to be responsible for controlling SV endocytosis kinetics and Syt1 retrieval in these neurons. However, in many inhibitory interneuron subtypes Syt2 is the dominant isoform (Chen et al. 2017). Intriguingly, alterations in short‐term synaptic plasticity were only observed in excitatory, but not in inhibitory neurons from SV2A/SV2B double knockout mice (Janz et al. 1999). However, cultured cortical inhibitory interneurons from the same mouse line did display a depression in evoked IPSCs and altered short‐term plasticity (Chang and Sudhof 2009). When the primary sequence of Syt2 is compared against the SV2A interaction site within the C2B domain of Syt1, there is a high degree of homology, suggesting that both should interact in a similar manner. However, whether this interaction is regulated in a similar manner is a key open question.

### 5.3 Does SV2A Control SV Endocytosis Kinetics via Syt1?

There is conclusive evidence that SV2A controls the retrieval and trafficking of Syt1 in a bidirectional manner. However, whether the altered trafficking of Syt1 results in a change in global SV endocytosis kinetics is still unclear. Increased surface stranding of Syt1 appears to facilitate SV endocytosis via the recruitment of PIPKIγ and dynamin‐1 (Bolz et al. 2023; Small et al. 2024). Furthermore, SV endocytosis is accelerated in the absence of stonin‐2 (Kononenko et al. 2013; Kaempf et al. 2015). However, whether SV endocytosis is accelerated by the surface stranding of Syt1 in the absence of SV2A is still unclear (Kaempf et al. 2015; Zhang et al. 2015). Regardless, is this regulation relevant to physiology, when one considers that the only instance this is observed is due to loss of function of stonin‐2 or potentially SV2A? It will be key to dissect with new structural mutations and measurement of dwell times on the plasma membrane to determine how Syt1 controls endocytosis kinetics during normal neuronal function.

### 5.4 Compartment‐Specific SV2A Interactions

Syt1 performs an essential role as the calcium trigger during activity‐dependent synchronous neurotransmitter release (Geppert et al. 1994). Therefore, an interaction with SV2A during this key stage of the SV life cycle may be deleterious to its primary function. This may explain why calcium negatively regulates the interaction (Schivell, Batchelor, and Bajjalieh 1996). However, it may also be the case that SV2A forms discrete complexes whether it is on SVs or the presynaptic plasma membrane. For example, a recent study that performed cross‐linking on a purified population of SVs revealed no interaction between SV2A and Syt1 (Wittig et al. 2021). In contrast, multiple interactions were observed between the lumenal domains of SV2A, synaptobrevin‐2 and synaptophysin. A key determinant may be SV2A phosphorylation, since this may determine priority of access for the Syt1 C2B domain. The fact that SV2A and Syt1 appear not to interact on SVs, suggest that its phosphorylation by casein kinase1 family kinases occurs exclusively at the plasma membrane. Therefore, it will be essential to reveal the subcellular localisation and activity‐dependence of this phosphorylation event, since it may be critical in determining how and when Syt1 retrieval complexes are constructed.

### 5.5 Do SV2A and Stonin‐2 Have Redundant Functions in the Control of Syt1 Trafficking?

The almost identical phenotypes relating to Syt1 targeting and retrieval produced by the loss of either SV2A or stonin‐2 suggest they may perform redundant functions. In support, both bind the C2 domains of Syt1 and share interactions with μ‐homology domains via their N‐termini; for SV2A via its tyrosine motif (Yao et al. 2010) and for stonin‐2 via multiple WVxF motifs (Walther et al. 2004). However, a key divergence is the lethality upon loss of SV2A in rodents (Crowder et al. 1999; Janz et al. 1999), compared to the apparent absence of phenotype in *Stn2* knockout mice which are viable and fertile (Kononenko et al. 2013). This may be due to redundancy with the related *Stn1* gene (Martina et al. 2001); however little is known regarding the function of this enigmatic gene (Maritzen, Podufall, and Haucke 2010). Furthermore, the impact of SV2A and stonin‐2 removal on Syt1 trafficking appears to be additive, suggesting complementary, but discrete mechanisms of action. Uncovering further molecular convergence and divergence between these two proteins will be critical in addressing this question.

### 5.6 How Does Loss of SV2A Function Cause Epilepsy?

Loss of function mutations in the human *SV2A* gene result in intractable epilepsy, however as discussed above, the mechanism underpinning this is still unclear. One potential explanation is a disproportionate impact on inhibitory neurotransmission, since SV2A appears to be more highly expressed at these synapses and is the only SV2 isoform (Bae et al. 2020; Bajjalieh et al. 1994). There are a few disparate stands of evidence that support this hypothesis. For example, while synapses from SV2A knockout mice routinely display no change in mEPSC or mIPSC frequency (Crowder et al. 1999; Custer et al. 2006; Chang and Sudhof 2009; Venkatesan et al. 2012), spontaneous IPSCs, reflecting evoked GABA release, are dramatically decreased (Crowder et al. 1999; Venkatesan et al. 2012). Furthermore, the speed of action of levetiracetam on supressing inhibitory neurotransmission is much faster compared to excitatory (Meehan et al. 2012). Intriguingly, many Syt1‐dependent presynaptic functions such as control of RRP size and clamping of spontaneous release only become sensitive to levels of Syt1 expression after 2 weeks in culture (Bouazza‐Arostegui et al. 2022). This delay was suggested to be potentially due to redundancy with other Syt isoforms such as Syt7, however this observation may also explain why SV2A mice begin to have fatal seizures between 2 and 3 weeks (Janz et al. 1999; Crowder et al. 1999).

## 6 Conclusions

The role that SV2A performs in presynaptic physiology and how its dysfunction links to epilepsy continues to be a matter of debate. However, a growing evidence base suggests that its interaction with Syt1 may be central to both roles. Future studies examining the key open questions outlined above will be critical in determining the function of this enigmatic molecule.

## Author Contributions

**James A. Hogg:** writing – original draft, writing – review and editing. **Michael A. Cousin:** funding acquisition, writing – original draft, writing – review and editing, supervision.

## Conflicts of Interest

Michael A. Cousin is a handling editor for the Journal of Neurochemistry.

### Peer Review

The peer review history for this article is available at https://www.webofscience.com/api/gateway/wos/peer‐review/10.1111/jnc.16308.

## Data Availability

Data sharing is not applicable to this article as no new data were created or analyzed in this study.
